# Retraction: Synthesis and application of monodisperse hydrophobic magnetite nanoparticles as an oil spill collector using an ionic liquid

**DOI:** 10.1039/d5ra90072g

**Published:** 2025-06-10

**Authors:** Ayman M. Atta, Abdelrhman O. Ezzat, Ahmed I. Hashem

**Affiliations:** a Chemistry Department, College of Science, King Saud University Riyadh 11451 Saudi Arabia aatta@ksu.edu.sa; b Petroleum Application Department, Egyptian Petroleum Research Institute Nasr City 11727 Cairo Egypt; c Chemistry Department, Faculty of Science, Ain Shams University Abasia 11566 Cairo Egypt

## Abstract

Retraction of ‘Synthesis and application of monodisperse hydrophobic magnetite nanoparticles as an oil spill collector using an ionic liquid’ by Ayman M. Atta *et al.*, *RSC Adv.*, 2017, **7**, 16524–16530, https://doi.org/10.1039/C7RA02426F.

The Royal Society of Chemistry, with the agreement of the authors, hereby wholly retracts this *RSC Advances* article due to concerns with the reliability of the data.

The TEM data in Fig. 4 has multiple repeating sections. The authors were not able to provide the original raw data nor satisfactorily explain the concerns.

Given the significance of these concerns, the findings presented in this paper are no longer reliable.

The authors were informed of the decision to retract this article. Ayman M. Atta has not agreed with the decision and the other authors have not responded.

Signed: Laura Fisher, Executive Editor, *RSC Advances*

Date: 28th May 2025

